# Identification and Assessment of Risks in Biobanking: The Case of the Cancer Institute of Bari

**DOI:** 10.3390/cancers14143460

**Published:** 2022-07-16

**Authors:** Giuseppe De Palma, Giulia Bolondi, Antonio Tufaro, Giuseppe Pelagio, Giuseppe Brando, Daniela Vitale, Angelo Virgilio Paradiso

**Affiliations:** 1 Institutional BioBank, Experimental Oncology and Biobank Management Unit, IRCCS Istituto Tumori Giovanni Paolo II Bari, Viale Orazio Flacco 65, 70124 Bari, Italy; a.tufaro@oncologico.bari.it (A.T.); g.pelagio@oncologico.bari.it (G.P.); a.paradiso@oncologico.bari.it (A.V.P.); 2 Marsh Advisory S.R.L., Viale Bodio 33, 20158 Milan, Italy; giulia.bolondi@marsh.com (G.B.); giuseppe.brando@marsh.com (G.B.); 3 Marsh S.p.A., Viale Bodio 33, 20158 Milan, Italy; danielaantonella.vitale@marsh.com

**Keywords:** biobanks, biological specimen banks, risk, risk management, quality, quality improvement

## Abstract

**Simple Summary:**

Risk assessment is one of the requirements for all activities involving the management of human biological samples within the framework of the new ISO 20387:2018. Although some theoretical approaches are available for preparing risk assessments in general, there is no evidence in the literature of examples of listed insurable risks for cancer biobanks. To fill this gap and to provide an overview of the survey performed in our cancer Biobank, we have assessed potential exposures to insurable risks. After an analysis of the Biobank structure and focusing on natural catastrophe risks, we produced a summary map of risk scenarios. In addition to implementing security awareness, this also lays the foundation for transferring the residual risk arising from Biobank activities to the insurance market.

**Abstract:**

Although research biobanks are among the most promising tools to fight disease and improve public health, there are a range of risks biobanks may face that mainly need to be assessed in an attempt to be relieved. We conducted a strategic insurance review of an institutional cancer biobank with the aim of both identifying the insurable risks of our own Biobank and gathering useful evidence of primary exposure to insurable risks. In this practical scenario, risks have been outlined and categorized into inherent and residual risks, along with their possible impact on biobank maintenance. Results at the Biobank of the Cancer Institute of Bari showed evidence of potentially significant and intrinsic risk due to highly relevant threats, along with already implemented improvements that significantly reduce risks to a range of relative acceptability.

## 1. Introduction

The effort of supporting a biobank also focuses on the ability to identify and assess risks, be prepared for disaster events and provide immediate solutions to preserve biological samples and data. Regardless of the type of security used, Biobanks are exposed to natural hazards (earthquakes, fires, floods), technical hazards (such as power outages) or damage caused by intrusions and theft of biological samples, computer hacking, loss of confidential data. As well as internal risks such as poor organization, internal accidental events, internal unlawful acts, failure to comply with procedures and errors/omissions by the Biobank staff. Sample and data security are undisputable and efficient risk management represents a must to avoid sample loss causing irreversible damage both to the Biobank as well as to the donors and stakeholders involved.

The issue cannot focus on “If the biobank can be affected by a disaster?” but on “Will the biobank be ready when a disaster occurs?” [1].

Today, biobanks are called to move forward from the certification scheme ISO 9001:2015 [2] to the ISO 20387:2018 accreditation scheme, called General requirements for biobanking [3,4]. This standard was developed with the objective of promoting confidence in biobanking. Furthermore, all of the procedures should ensure *address risks and opportunities* using a risk assessment. Options to address risks include, first of all, identifying and avoiding threats [5].

Biobanking is also characterized by a large number of non-binding regulations that result in self-assessed practices to manage associated risks [6].

It is difficult to outline and implement a crisis management plan. Parry-Jones et al. [7] provided a general outline for the development of such a plan for risk analysis and management.

Recently, based on these criteria, the Biobank team of the IRCCS Cancer Institute “Giovanni Paolo II”, supported by the Marsh insurance brokerage company (www.marsh.com/it/en/home.html) (accessed on 17 May 2022) and its subsidiary, Marsh Advisory (a consulting company specializing in risk management services), recently drew up a Risk Assessment & Strategic Insurance Review, identifying and evaluating insurable risks for use in BioBank management. The results are reported in this article and many of the scenarios covered are applicable to other cancer biobanks.

## 2. Materials and Methods

### 2.1. Biobank Characteristics

The BioBank is located in a facility consisting of a mezzanine and a basement. In the basement, there are two cryostorage rooms. On the first floor, there are two laboratories, connected by a pass-box, one for the preparation of biological resources for research purposes (classified BSL-2) and the other for the reception, processing and sorting of biological samples (classified BSL-2) along with the management office.

A 10,000 L external cryogenic liquid nitrogen tank is connected to the BioBank through a super-insulated stainless steel vacuum line for backup and refill of four LN2 cryogenic freezers, nine −80 °C ultra-freezers and a controlled rate freezer. There is air exchange system that guarantees both maintenances of constant temperature and humidity and continuous air exchange. Power failure is prevented by an uninterruptible power supply (UPS) system and the Biobank is protected by an alarmed anti-intrusion system monitored and controlled 24/7 by dedicated personnel. Unauthorized access is guaranteed by use of personal RFID badges, PIN codes and fingerprint scans. Internal video surveillance is fully recorded and connected with the National technical assistance center which provides prompt remote or on-site intervention. Sample data and storage conditions are managed by CryoSMART™ (Air Liquide Sanità Service Spa, Milan, Italy) a dedicated and custom-tailored software with full traceability and GLP validation. Any critical alert is sent via SMS to the Biobank team enabling immediate intervention.

Biobank management consists of a coordinator and a scientific technical board (STB), supported by a board of researchers along with a stakeholder committee.

The BioBank is certified UNI EN ISO 9001:2015 to ensure strict compliance with quality in the management of processes relating to the collection, processing, storage and distribution of biological samples of human and animal origin, focused mainly on cancer, but also on other diseases for use in scientific and research activities.

**Figure 1:**
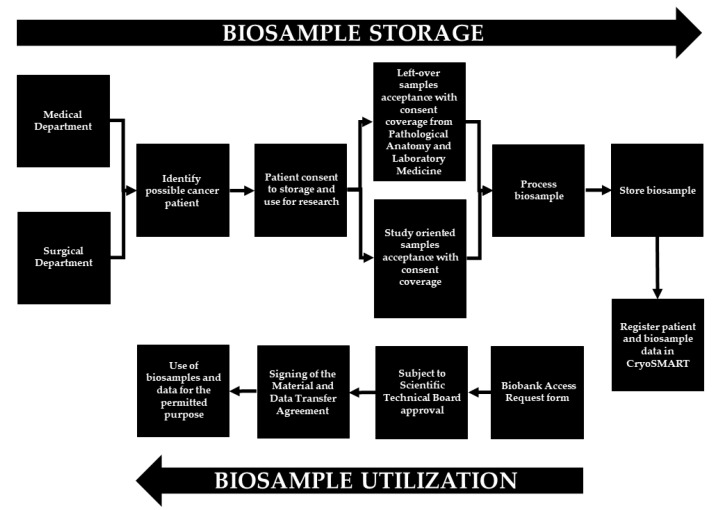
Bari Biobank biosample workflow. The arrow to the right of the figure shows the processes progression designated for the biosample storage. The arrow to the left of the figure shows show those for the biosample utilization.

Figure 1 maps the general biobanking operational activities for study-oriented and left-over biosamples.

Currently, the Biobank collects and stores biosamples from over 50,000 patients, with an average of ∼10,000 stored aliquots per year, with clinical and pathological data and donor consent and manages collection, processing, storage and distribution of samples including collaboration in over 40 clinical studies related to cancer.

### 2.2. Operational Approach

The operational approach to risk assessment process was initially based on collection and analysis of documentation; a series of preliminary meetings for definition of the methodological framework (i.e., risk model, assessment criteria were also held).

Following interviews, during November 2019, both with the staff and the coordinator of the Biobank, and with the data protection officer, the IT manager and the general manager of the cancer institute (i.e., risk owners), we were able to identify and evaluate risks and controls in place for each process. The identified risks were assessed considering the probability of occurrence (on a scale from 1 to 5: 1—Rare, 2—Unlikely, 3—Possible, 4—Likely, 5—Very likely) and the potential impact (on a scale from 1 to 5: 1—Negligible, 2—Minor, 3—Moderate, 4—Serious, 5—Critical) based on available data on historical claims and projections, with the result of defining the inherent risk.

In addition, the controls in place to reduce the probability (preventive controls), the impact (corrective controls) or both the probability and the impact (combined controls) of the identified risks were mapped, and their effectiveness was evaluated (on a scale from 0 to 5: 0—Absent; 1—Very weak; 2—Weak; 3—Moderately effective; 4—Effective; 5—Very effective) by the risk owners according to their experience and based on the available data and projections regarding the following two dimensions: the presence and degree of implementation of control mechanisms, procedures and organizational solutions; the adequacy of the IT infrastructure and the technological systems.

To obtain the residual probability and the residual impact, a reduction factor (i.e., RF) depending on the effectiveness of the controls (see Table 1) was employed according to the following formula: in case of preventive controls, Residual Probability=Inherent Probability×RF; in case of corrective controls, Residual Impact=Inherent Impact∗RF; in case of combined controls, Residual Impact=Inherent Impact×((1+RF)/2) and Residual Probability=Inherent Probability×((1+RF)/2).

Finally, the residual risk was obtained as the product between the residual probability and the residual impact.

All the information was collected in the risk register, and the results were aggregated and prioritized. A specific analysis was carried out through the identification of mitigation solutions on an organizational, procedural, contractual and technical basis for the top risks identified.

We have also considered and evaluated natural disasters as a variable that can endanger the integrity of biological specimens, using an online natural disaster risk map system produced by Munich Re called NATHAN [8], created to estimate the risk of various natural disasters around the world.

## 3. Results

### 3.1. Natural Disaster Risk

According to NATHAN, the exposure risk to natural disaster is low. The most likely are flash floods, fulmination, and earthquakes. The ground floor of the Biobank is about 70 cm below street level, while access to the basement is via an external staircase. Flooding risks are prevented by means of draining wells and a sump pump in the basement. Minor flooding is channeled into the sewerage system. The Biobank is also protected against lightning according to International Electrotechnical Commission (IEC) standard IEC 62305-2:2010 and has been constructed according to the prevention of damage due to the boundaries in case of seismic activity.

### 3.2. Significant Inherent and Moderate Residual Risks

#### Compromising of Confidentiality/Integrity/Availability of Personal Data

The privacy of donors is, in all cases, guaranteed by the EU GDPR Institutional Code of Conduct officially approved by the General Director Act n.2100/2019. The Act defines the rules for compliance with principles of fair and transparent processing.

Biobank activities concern the storage of the following: biosamples ad hoc collected within specific clinical protocols; left-over material from standard clinical practice.

In the first case, the autonomy in the management of biosamples is strictly detailed in informed consents of various protocols and signed time-to-time by the donors. Informed consent utilized in various protocols always specifies as follows: the management of possible incidental findings related to specific laboratory investigations approved by the ethical committee (EC); management of samples from deceased, children or people under legal tuition. For samples collected within specific trials EC approved, the management of samples and data are study specific and always limited to the objectives of the protocol.

In our BioBank, donor and clinical data, classified as sensitive according to the EU General Data Protection Regulation 2016/679, is stored in a dedicated management and control system called CryoSMART™. This management system is designed to comply with the US Food and Drug Administration’s 21 CFR Part 11 regulations and all data management procedures operate in compliance with ISO/IEC 27001:2013. The software runs on a dedicated server that is independent of that of the Institute. There is full segregation both at the network level and at the physical structure level. The data is transferred to researchers mostly in fully anonymized or pseudo-anonymized format (the Biobank provides an identification code that can be decrypted only by authorized Biobank staff).

The scenario may occur and with a potentially severe impact, although the controls in place by the IT manager to protect the infrastructure against cyber-attacks and by omitting parts of the data that are not required for research (principle of data minimization) reduce both the risk’s likelihood and impact.

### 3.3. Moderate Inherent and Moderate Residual Risks

#### 3.3.1. Unusability of Cryopreserved Biological Material

This scenario includes damage to biological material intended for research. Standard refrigerator-freezers, ultra-freezers and cryogenic freezers cooled by liquid nitrogen must have a constant temperature to allow cryopreservation. The main risk is ultra-freezer failure due to a prolonged power outage, as most of our samples are stored in these freezers. As well as valuable biosamples might be lost because of the prolonged interruption of the liquid nitrogen supply.

The time between collection and cryopreservation, in the case of fresh tissue, should not be more than 60 min (warm or cold ischemia). During this period, the material must be quickly transported to the BioBank, preferably on cold ice, and prepared for cryopreservation. If the process exceeds the suggested time, the sample may not be suitable for particular procedures or may be no longer usable. In some cases, the quality of the sample is reviewed by the pathologist to assess whether or not to proceed with cryopreservation or use it for certain analyses. If this occurs, the sample shall be classified as “not usable” or “partially usable”. The percentage of unusable biospecimens in 2020 has been 2.08% due to the longer sample transfer time to the biobank. Controls in place (i.e., protocols and procedures, technical instruments etc.) maintain this scenario in a moderate risk area. In fact, CryoSMART, thanks to the connection of all critical alarms, allows the sending of alarms to Biobank staff, available 24 h a day and 7 days a week, via the web, mail, voice call and message. In addition, the procedure provides that the Biobank staff monitor the pressure and level parameters of the external liquid nitrogen tank on a daily basis to prevent emptying. Furthermore, with a view to continuous improvement of internal processes, the monitoring of collection and cryopreservation times was defined. These data are analyzed every semester.

#### 3.3.2. Accidents of Researchers, Employees or Technicians in Laboratories and Cryogenic Rooms

This scenario includes accidents (mild or severe) that may occur in laboratories or cryopreservation areas. Accidents may affect BioBank personnel but also Cancer Institute employees who access the BioBank. Prevention measures in place (i.e., protocols and procedures, training activities, quality and safety of technical instruments, security systems, emergency procedures etc.) mitigate the risk.

### 3.4. Significant Inherent and Slight Residual Risks

#### 3.4.1. Infringement of the Rules on the Conservation of Biological Material

For biological left-over samples, BioBank staff can proceed to sample procurement and storage only if the sample itself is accompanied by a copy of the informed consent form. The Biobank has adopted a multioption consent where partial restriction can be chosen by the donor, as well as the right to withdraw consent for samples and associated data storage at any time, also by close relatives in case of inability or death.

In specific, donors sign a standard Informed Consent permitting the future utilization of biosamples for cancer research purposes in other national and international research institutions after the approval of STB and authorization of EC. For specific projects, biosamples can also be moved to private companies after previously already described approval steps. The informed consent foresees the possibility to deny the authorization of utilization outside the Institute, outside Italy, outside public research Institutions.

In the case of biosamples from children under age 18, or donors with disabilities, consent must be provided by a parent, spouse or legal guardian. In all cases, biosample donors always declare if they prefer to be contacted to be informed about utilization or clinical-biological data concerning their biosamples.

If the copy of the informed consent form is not transferred to the biobank, the biobank staff will check whether or not the signed original is contained in the donor’s medical records. If not, the collected material shall be immediately disposed of.

A copy of informed consent was acquired and registered in 93.94% of cases in the last 3 years. Reasons for consent unavailability were forgetfulness or lack of time for clinicians involved in routine priority activities. Considering the potential impact of the risk, the inherent severity is significant; however, the controls in place mitigate the risk as follows: at the beginning of each semester, the Biobank Data Manager at the beginning of each semester, the Biobank Data Manager informs the pathologist and the clinician referent of the percentages of cases lacking informed consent.

#### 3.4.2. Prolonged Interruption of BioBank Activities (without Compromising the Biological Material)

Prolonged interruption of BioBank activities can be attributed to insufficient storage space, software inefficiency or BioBank staff unavailability. This staff, at this time, consists of one manager, two researchers and one technician. Staff reduction obviously represents a relevant issue for BioBank activities, resulting in the loss of personnel with expertise developed over many years with consequential difficulty in recruiting new personnel with experience in biobanking. Considering the other possible causes, BioBank has implemented mitigation measures that consent to reduce the significance of the risk as follows: redundancy of the spaces and a service level agreement with the software supplier.

### 3.5. Moderate Inherent and Slight Residual Risks

#### 3.5.1. Compromise of the Value of the Biological Sample

The value of the biological sample is estimated on the basis of the information associated with the sample and its clinical and pathological characteristics, as well as the rarity of the disease. Poor or missing associated sample data may result in a sample value reduction when performing research. BioBank donor consent clarifies that sample and data biobanking, use and distribution is fully non-commercial, although cost recovery for sample management is required.

#### 3.5.2. Loss of Data Related to Samples

This scenario includes the loss of data caused by unlawful actions by the Biobank team or by accidental events caused by them as a result of an error in the performance of their duties or an external force majeure event. The likelihood of this risk is low and the impact could be significant, but the controls in place maintain the scenario in a slight residual risk area as follows: our data management system automatically generates a daily encrypted database backup, both on a physical Biobank server and on a cloud storage server.

## 4. Discussion

Governance of a biobank is also important as follows: it must try to find ways to recognize and balance both the risks associated with the use of the data and the counterfactual harms associated with not using the data that could otherwise produce collective benefits [9].

One of the risks associated with biobanking is the violation of donor confidentiality and the consequent loss of privacy. Biobanking can therefore have negative effects on the donor’s insurance policies and job opportunities [10]. Many donors are aware that there are risks associated with biobank participation associated not only with sampling but also with data management such as privacy breaches. They are, however, open to collaboration [11,12]. From an ethical and legal standpoint, our Biobank has chosen the participation with a return of incidental findings. This level of participation allows the donor/family to receive information regarding individual, clinically/medically relevant information identified through research with the stored samples [13].

**Figure 2:**
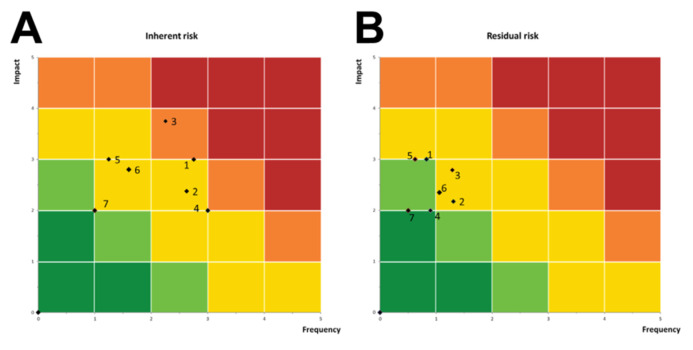
The Figure present the obtained risk scenarios according to their inherent risk ((**A**), left matrix) and residual risk ((**B**), right matrix) profile. Impact of risks is plotted on the y-axis while frequency of risks is plotted on the x-axis. Both use a one to five scoring scale with one being the lowest and five being the highest. (1) Impaired confidentiality; (2) Unusability of cryopreserved biological material; (3) Accidents of researchers, employees or technicians in laboratories and cryogenic rooms; (4) Infringement of the rules on the conservation of biological material; (5) Prolonged interruption of BioBank activities; (6) Compromise of the value of the biological sample; (7) Loss of data related to samples.

Security is still a neglected issue in the debate on biobanking that however has seen, in recent years, methodological and technological developments both for the amount of sample and for data collected and shared globally [14]. There are several issues involved in Biobank risk management regarding infrastructural-technical and personnel security, biobanking practice and data protection [15]. Impact assessment plays an important role, especially regarding the challenge of the creation of properly informed consent templates for biobank-related research [16]. Our assessment underlines that measures must be put in place to address the risks arising from data processing of sensitive personal data, and the sharing of such data with third-party [17]. It is useful to counter foreseeable risks. Both ongoing commitments inside and outside the biobank are essential to support a critical risk assessment that can adapt to developments [18]. Using the SWOT analysis as a risk management tool, Sargsyan et al. have determined the strengths, weaknesses, opportunities and threats of two emerging Biobanks at the National Institute of Public Health of Kosovo and the University of Tirana Medicine. On the basis of this analysis, they then defined more efficient strategies for biobanking processes and the use of existing infrastructures and knowledge in research [19]. Miranda et al. summarize the potential risks and control measures associated with the acquisition of human samples in research identified in projects under the remit of the UCL/UCLH Biobank for Studying Health and Disease of University College London Cancer Institute at London, as well as transportation, use, storage and disposal of human samples and the security of the premises [20]. However, thanks to the collaboration with Marsh and Marsh Advisory, we have explored threats and risks, however remote it may seem. In this article, in light of the considerable specificity that ISO 20387:2018 requires a risk assessment, we have tried to briefly examine the most important risk scenarios at the present. With inherent and residual risks assigned, scenarios can now be plotted on the 5 × 5 risk matrix. The risk profile that emerged as illustrated by the risk matrix in Figure 2, indicates that the Biobank presents a potentially significant inherent risk, with probable and highly material threats, but at the same time the implemented control solutions significantly reduce the risks and bring them back to an area of relative acceptability. The next step, in the light of this rigorous analysis, will be to discuss with insurance companies interested in insuring Biobank activities are represented in the yellow zone in Figure 1B indicating that risk is at the moderate level. Even more important will be to check for the application to artificial intelligence approaches to management of our biobank. In the next future, Biobanks will play a central role in producing metadata from large collections of high-quality well-annotated samples thus requiring AI approaches to permit efficient data management solutions [21]. In this perspective, main efforts are now dedicated to the organization of technological infrastructures able to store biosamples information, medical images and clinical data [22]. In particular, Aibibank supported by Piemonte Region is exploring the possibilities to manage biobank-metadata production by AI and Deep Learning approaches [23]. It is worth noting that the program of Dubai Universit is realizing a new robotic biobank moved by an automated artificial intelligence tool [24].

## Figures and Tables

**Table 1:** The Table presents the evaluation scale, the descriptions and the reduction factors that were attributed to each control.

Scale	Evaluation	Description	Reduction Factor
**5**	Very effective	- There are control mechanisms/procedures/appropriate organizational solutions and they are always applied - There are adequate technological/IT systems	0.1
**4**	Effective	- There are control mechanisms/procedures/organizational solutions that are adequate but not always applied - There are mainly adequate technological/IT systems	0.3
**3**	Moderately effective	- There are partially adequate control mechanisms/procedures/organizational solutions and they are always applied - Partially adequate technological/IT systems exist	0.5
**2**	Weak	- There are control mechanisms/procedures/organizational solutions partially adequate and not always applied - There are mainly inadequate technological/IT systems	0.7
**1**	Very weak	- There are inadequate control mechanisms/procedures/organizational solutions - There are inadequate technological/IT systems	0.9
**0**	Absent	- There are no safeguards/interventions to prevent/mitigate the risk	1

## Data Availability

The data presented in this study are available on request from the corresponding author. The data are not publicly available because are propriety of IRCCS Istituto Tumori "Giovanni Paolo II" Bari.
